# Analyses of cerebral microdialysis in patients with traumatic brain injury: relations to intracranial pressure, cerebral perfusion pressure and catheter placement

**DOI:** 10.1186/1741-7015-9-21

**Published:** 2011-03-02

**Authors:** David W Nelson, Björn Thornquist, Robert M MacCallum, Harriet Nyström, Anders Holst, Anders Rudehill, Michael Wanecek, Bo-Michael Bellander, Eddie Weitzberg

**Affiliations:** 1 Section of Anesthesiology and Intensive Care, Department of Physiology and Pharmacology, Karolinska Institutet, Stockholm, Sweden; 2 Division of Cell and Molecular Biology, Immunogenomics Group, Imperial College London, London, UK; 3 Section of Neuroradiology, Department of Clinical Neuroscience, Karolinska Institutet, Stockholm, Sweden; 4 Swedish Institute of Computer Science (SICS), Kista, Sweden; 5 Section of Neurosurgery, Department of Clinical Neuroscience, Karolinska Institutet, Stockholm, Sweden

## Abstract

**Background:**

Cerebral microdialysis (MD) is used to monitor local brain chemistry of patients with traumatic brain injury (TBI). Despite an extensive literature on cerebral MD in the clinical setting, it remains unclear how individual levels of real-time MD data are to be interpreted. Intracranial pressure (ICP) and cerebral perfusion pressure (CPP) are important continuous brain monitors in neurointensive care. They are used as surrogate monitors of cerebral blood flow and have an established relation to outcome. The purpose of this study was to investigate the relations between MD parameters and ICP and/or CPP in patients with TBI.

**Methods:**

Cerebral MD, ICP and CPP were monitored in 90 patients with TBI. Data were extensively analyzed, using over 7,350 samples of complete (hourly) MD data sets (glucose, lactate, pyruvate and glycerol) to seek representations of ICP, CPP and MD that were best correlated. MD catheter positions were located on computed tomography scans as pericontusional or nonpericontusional. MD markers were analyzed for correlations to ICP and CPP using time series regression analysis, mixed effects models and nonlinear (artificial neural networks) computer-based pattern recognition methods.

**Results:**

Despite much data indicating highly perturbed metabolism, MD shows weak correlations to ICP and CPP. In contrast, the autocorrelation of MD is high for all markers, even at up to 30 future hours. Consequently, subject identity alone explains 52% to 75% of MD marker variance. This indicates that the dominant metabolic processes monitored with MD are long-term, spanning days or longer. In comparison, short-term (differenced or Δ) changes of MD vs. CPP are significantly correlated in pericontusional locations, but with less than 1% explained variance. Moreover, CPP and ICP were significantly related to outcome based on Glasgow Outcome Scale scores, while no significant relations were found between outcome and MD.

**Conclusions:**

The multitude of highly perturbed local chemistry seen with MD in patients with TBI predominately represents long-term metabolic patterns and is weakly correlated to ICP and CPP. This suggests that disturbances other than pressure and/or flow have a dominant influence on MD levels in patients with TBI.

## Background

Cerebral microdialysis (MD) has been used to monitor patients with traumatic brain injury (TBI) for over a decade, but the methodology has not yet found a clear place in the neurointensive care unit (NICU) arsenal of multimodal monitoring [1,2]. The commonly monitored parameters that are advocated to follow dynamic metabolic changes in viable but vulnerable tissue (and their current predominant interpretations) are lactate, pyruvate (metabolic markers of redox state and thus ischemia and/or hypoxia), glucose (local capillary flow, but also related to blood glucose and metabolism), glutamate (excitotoxic marker) and glycerol (phospholipid degradation as a marker of cell breakdown and death) [3]. Baseline values have been investigated [4], and ischemic interpretations of MD have been suggested [5] and are supported by findings from the ischemic penumbra [6]. MD has been shown repeatedly to correlate with other components of multimodal brain monitoring, such as jugular venous saturation and brain tissue oxygenation [3]. Specifically, intracranial pressure (ICP) and cerebral perfusion pressure (CPP) have both been reported to correlate with MD values [7,8], and manipulation of these parameters are often first-line bedside responses to pathological MD values. CPP has also been claimed to be the most frequently used surrogate monitor of cerebral blood flow [9].

Despite the discussion above, it remains unclear how the individual levels of the real-time MD data streams are to be interpreted on a patient-to-patient basis, and the value of using MD in the treatment of TBI has not yet been established [2,3,10,11]. While MD-derived data seem to be a sensitive monitor of local ischemic tissue, as shown especially in the experimental ischemic penumbra [12], there is a growing awareness that the classical "ischemic" interpretation of MD values in the traumatic border zone may often reflect metabolic states unrelated to ischemia or tissue hypoxia [13]. In addition, the scope of ischemia in TBI may not be as extensive as previously thought [13,14]. Although sensitive, MD may thus be a nonspecific monitor of ischemia [11]. This appears to be in conflict with the expectations of MD as a dynamic monitor of ischemia used for online interpretation and decision-making.

The real-time interpretability of an online monitoring system is fundamental for its use, but conflicting interpretations of MD in TBI have emerged, especially those that can potentially be interpreted as ischemia and/or hypoxia. The concept of ICP- and CPP-vulnerable pericontusional tissue, where MD could be used to monitor the dynamic metabolic effects of local oxygen delivery-dependent ischemia and/or hypoxia, contrasts with that of possible cytopathic hypoxic "states." Here, instead, local oxygen utilization itself could be altered, such as by mitochondrial dysfunction [15] or diffusion barriers [16]. These states would be expected to be less susceptible to ICP and CPP variation. A consensus [17] and an investigation [18] have also highlighted the importance of computed tomography (CT)-verified catheter placement, and MD is suggested to be most informative when monitoring vulnerable, ischemia- and/or hypoxia-prone, traumatic pericontusional (border zone) tissue.

The analysis of MD data requires special consideration, as it is a time series, where intrapatient data hours are correlated. This must be taken into account in analysis. We have found earlier in TBI patients that by using a computer-based pattern recognition method (self-organizing maps) [19], MD patterns, even those potentially interpretable as ischemia, were unexpectedly static over time and therefore highly delineate subjects. This suggests that the dominant monitored metabolic processes with MD were long-term, extending over whole patient-monitoring periods. It has been recognized that these data regarding intrapatient time dependency have often been overlooked in the analysis of MD data [20]. Despite this, hourly MD from individuals continues to be analyzed as independent data [21,22].

The aim of the present study was to establish, in a large MD data set, the extent to which MD correlates to ICP and CPP when taking into account the correlated time series nature of MD data in analyses. A strong correlation of MD values to ICP and CPP would suggest pressure and/or flow processes to be central causes of locally perturbed metabolism. To this purpose, extensive data mining was performed. In line with consensus, a CT-defined group of optimally placed pericontusional catheters was identified and contrasted with nonpericontusional placed catheters. In addition, MD, CPP and ICP data were analyzed for their relation to Glasgow Outcome Scale (GOS) score.

## Methods

The study was approved by the local ethics committee on human research at the Karolinska Institutet and the Karolinska University Hospital. Our standard NICU care was applied, and no interventions were employed as part of this investigation.

### Inclusion

This study was a retrospective analysis including all consecutive patients admitted to the adult (≥15 years of age) NICU with TBI requiring mechanical ventilation (generally Glasgow Coma Scale (GCS) score ≤ 8), during a 5-year period, with functioning MD catheters, ICP monitoring and arterial catheters.

### Patient management

All patients were intubated, mechanically ventilated and sedated with morphine, midazolam or propofol. Mass lesions were evacuated as deemed appropriate by neurosurgeons. ICP was measured predominantly with ventricular catheters or, in some cases, with intraparenchymal pressure monitors (Codman & Shurtleff inc. Raynham, MA, USA). Mean arterial pressure (MAP) was measured invasively, commonly in the radial artery. CPP was calculated as MAP-ICP, with both transducers placed at the midlateral ventricular level. Patients' heads were elevated at 20°to 30°angles. ICP was targeted at ≤ 20 mmHg and CPP was targeted at 60 to 70 mmHg. Targets were achieved with intravascular infusions (Ringer's acetate and albumin), vasopressors (norepinephrine), osmotic therapy (hypertonic NaCl and mannitol), intermittent cerebral spinal fluid (CSF) drainage from ventricular catheters, ventilation and temperature control, and decompressive craniotomy as needed. When ICP could not be controlled with other measures, sodium thiopental was infused, limited by burst suppression and monitored with continuous electroencephalography. Partial pressure of carbon dioxide was targeted at 4.5 kPa. When mild hyperventilation was employed for ICP control, it was guided by venous jugular bulbar saturation and arterial-jugular lactate difference. Temperature was regulated at 37°C with paracetamol or external wrapping cooling systems. Mild hypothermia (35°C to 36°C) was used for high refractory ICP. Blood glucose was targeted at 4 to 8 mM/l, and hemoglobin was targeted at ≥90 g/l.

### Microdialysis technique

CT-visible gold-tipped MD catheters of 10-mm length and a 20-kDa cutoff CMA 70 (Solna, Sweden) were placed in conjunction with evacuation of mass lesions or placement of ICP monitors in the surgical theater. Catheters were perfused with a solution with an electrolyte composition similar to the CSF (CMA) at 0.3 μl/min via a pump (CMA 106). Dialysate was sampled in vials and analyzed immediately for glucose, lactate, pyruvate and glycerol levels using the CMA 600 enzyme photometric analyzer at 1-hour intervals. The extraction ratio of this catheter and perfusion rate is known to be close to 70% [23]. MD catheters were viewed on CT scans with the assistance of a neuroradiologist. Pericontusional location was defined as within 2 cm of a mass lesion (hyperdense or hypodense contusion or a hematoma border) on CT scans. Catheters not fulfilling this definition were defined as nonpericontusional.

### Data acquisition and preparation

ICP and MAP data were collected at 1- to 2-minute intervals with the Datex monitoring system (Datex-Ohmeda, Helsinki, Finland) and saved to a computer disk with MD data using the program ICU pilot (CMA). Data were checked for integrity, removing known artifacts such as arterial catheter flushing, nonfunctioning dialysate pumps, catheters producing empty vials or logged events of erroneous handling or labeling of vials. A MD catheter transfer time (from membrane to vial) of 17 minutes was adjusted for. Limiters were applied to out-of-range data (as specified by CMA) to avoid nonsensical ratios. Sets of complete markers (glucose, lactate, pyruvate and glycerol) were extracted with ICP and/or CPP and time codes. Ratios of lactate:pyruvate (LP ratio) and lactate:glucose (LG ratio) were calculated. MD data were viewed for skew, and standard log_10 _and square-root transformations were performed to approach normal distributions (lactate, pyruvate and glucose, square root; glycerol, log_10_). Glasgow Outcome Scale (GOS) scores [24] were recorded at three time points: neurosurgical discharge, 3 to 6 months posttrauma and ≥1 year posttrauma. The best GOS score was defined as the highest value of these time points and was used in the study. We hypothesize that this value is most related to structural TBI changes. Patients with only discharge GOS scores and those with GOS scores from 2 to 4 were considered lost to follow-up.

### Statistical analyses

Analyses were performed using MATLAB (MathWorks, Natick, MA, USA) and the statistical program R (R Foundation for Statistical Computing, Vienna, Austria; http://www.R-project.org) [25].

### Autocorrelation and cross-correlation of MD, ICP and CPP data

Our earlier study suggested predominantly long-term (on the order of days) patterns of MD and thus that there should be a high autocorrelation [19]. Therefore, MD and ICP and/or CPP were analyzed for autocorrelation and cross-correlations (per patient) of the time series. Autocorrelation is the extent to which values are correlated with themselves over time. Cross-correlation refers to the extent to which variables are correlated with each other. In the cross-correlation analysis, checks were also performed using (per patient) randomly permuted MD data, thus eliminating the time series component of the data.

### Multivariate correlations of MD, ICP and CPP data

Composite patterns of MD may be more related to ICP and/or CPP than individual markers. Multivariate analysis was therefore employed using two methods (one linear method, mixed effects linear models; and one nonlinear method, artificial neural networks (ANNs)). Both avoid the principal problems of intrasubject data dependency, but by different means. With mixed effects linear models, the autocorrelated structure of the data can be accounted for in analyses, and significant correlations in excess of this can be evaluated. With ANNs, a cross-validation procedure is employed (in our case leaving out one patient at a time) to ensure that correlations generalize and are thus relevant to all patients.

Mixed effects linear models were fitted using Restricted Maximum Likelihood (REML) using the NMLE library for R. Random effects were those of patients, with an autoregressive moving-average covariance (ARMA (1,1)) structure for near lying data hours.

ANNs with radial basis functions were trained to predict ICP and/or CPP from MD values. The strength of predictions was assessed as the correlation of true vs. predicted ICP and/or CPP. The optimal number of nodes and training epochs as well as model assessments were determined using cross-validation (Radial Basis Networks, MATLAB ANN module, Netlab by Bishop http://www1.aston.ac.uk/eas/research/groups/ncrg/resources/netlab/).

Hourly means of ICP and CPP (hour prior to MD sample time) were the predicted variables. An extended search was also performed to identify other possible representations or time points of ICP and/or CPP (hour or percentage of monitoring over or under cutoffs) that could enhance predictions.

As a consequence of findings from autocorrelation and cross-correlation analyses, mean (per patient) MD, ICP and CPP data were subsequently correlated using univariate and multivariate analysis. Outliers giving undue influence to correlations were identified as Cook's distance > 1.

### Relative changes of MD data toward ICP and/or CPP

Pertinent information may be found in short-term (hourly) changes of MD. Univariate and multivariate correlations of relative levels of MD toward ICP/CPP were explored by two methods using linear regression. First, Δ values, differentiated MD (from 1- to 4-hourly samples) were correlated to ICP and/or CPP (absolute levels and differentiated). Second, individual normalization (that is, relative changes of a patient's MD values around their own means), which is justified if there are differing "baseline" values for local areas where MD catheters are positioned (and thus for patients), and short-term changes are superimposed on these baselines. Patients' data were normalized to a mean of zero and standard deviation of 1. Data were also checked to identify any need for detrending (relative values around a general trend or baseline, such as that patients generally get better over time).

### Outcome analysis

One-way analysis of variance (ANOVA) and the Kruskal-Wallis test were used for analyses of MD and ICP and/or CPP toward GOS score. Patients with less than 12 hours of MD were excluded from outcome analyses.

## Results

### Data description

Ninety patients were eligible for analysis and ranged in age from 15 to 77 years. The patients' mean age was 48.9 years. The mean GCS score was 6.5 (median, 6.0). Admission GCS score ranges were 3 to 8 (72%), 9 to 12 (18%) and 13 to 15 (10%). Complete sets (all four markers) of MD were obtained for a mean of 84 (hourly taken samples) per patient. Sixty-four catheters (64 patients, 5,645 complete MD samples) were identified as pericontusional on the basis of CT scans, and 26 (26 patients, 1,731 complete MD samples) were identified as nonpericontusional. One patient was lost to follow-up and had only a GOS score at discharge. Five patients had less than 12 hours of MD and were excluded from outcome analysis. Mortality was 15%, and there was a 57% percent favorable outcome (GOS scores 4 and 5). No patient was vegetative (GOS score 2).

### Pooled MD data in relation to catheter placement, ICP and CPP

**Figure 1:**
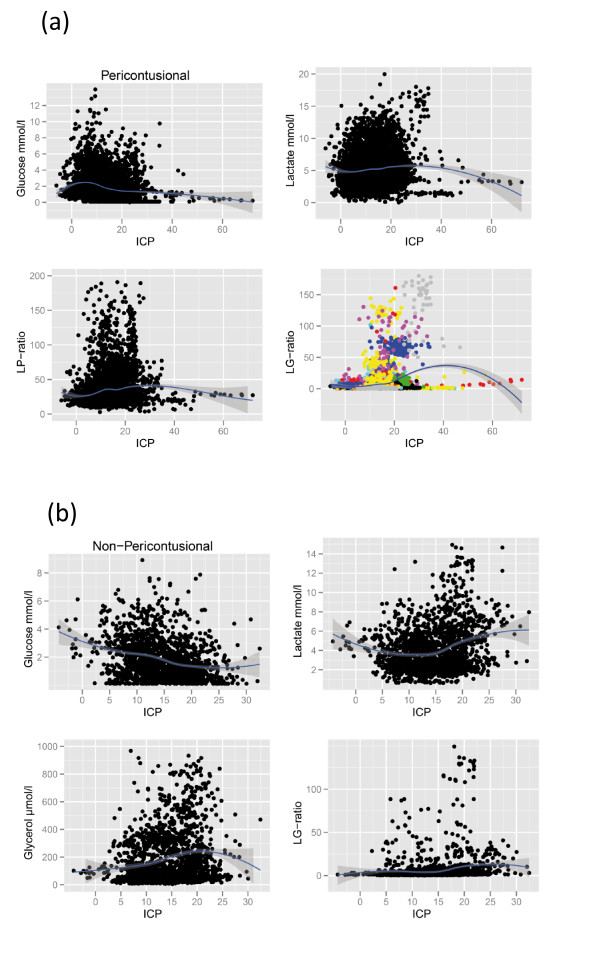
**Scatterplots of intracranial pressures (ICP) vs. microdialysis (MD) markers**. Markers selected are those with possible trends in Table 1. A locally fitted regression (lowess) is supplied with a shaded standard error. A colored graph is shown to illustrate how individuals cluster and thus disproportionally affect binned data. **(A) **MD from pericontusional located catheters. **(B) **MD from nonpericontusional located catheters. Lactate:pyruvate (LP) ratio, lactate:glucose (LG) ratio.

**Figure 2:**
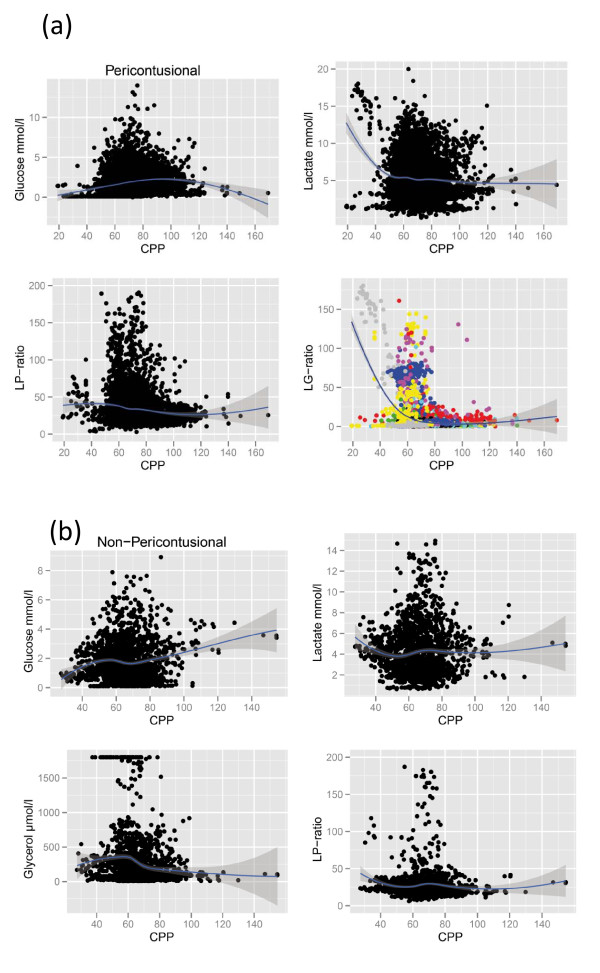
**Scatterplots of cerebral perfusion pressures (CPP) vs. microdialysis (MD) markers**. Markers selected are those with possible trends in Table 2. A locally fitted regression (lowess) is supplied with a shaded standard error. A colored graph is shown to illustrate how individuals cluster and thus disproportionally affect binned data. **(A) **MD from pericontusional located catheters. The colored lactate:glucose (LG) ratio scatterplot indicates that one patient is mainly responsible for the apparent LG ratio threshold at low CPPs found in Table 2. The intersubject reliability of this MD response therefore cannot be judged on the basis of these data. **(B) **MD from nonpericontusional located catheters. Lactate:pyruvate (LP) ratio.

Mean MD data from pericontusional and nonpericontusional catheters are shown in Tables 1 and 2 and binned to ICP and CPP intervals, respectively. Extreme caution must be observed when interpreting such tables with pooled values, as they do not take into account the correlated nature of repeated measures within subjects. A patient's data will be unevenly distributed between the bins and can thus affect bins disproportionally. Despite this, primarily glucose levels may suggest an interesting, and possibly expected, trend in relation to ICP and CPP levels. Another approach to visualizing these data is a scatterplot with a nonlinear fit (lowess locally weighted regression) (Figures 1 and 2). The shown MD markers chosen are displaying possible trends in Tables 1 and 2. The *y*-axes for the LP ratios are truncated to allow better inspection of the plots around the regression line, not showing some extreme high values, but all data were used for the lowess fit. The data below 50 mmHg for CPP must be judged (as above) with caution, as the extreme MD values in this region are seen clustered mainly from one subject and the consistency of MD responses at this cutoff between subjects can therefore not be evaluated.

**Table 1:** Pooled microdialysis data and intracranial pressurea^a^

			ICP, mmHg		
	
Microdialysis data	< 15	15 to 20	20 to 25	25 to 30	> 30
Pericontusional					
Glucose, mM/l	2.2 ± 1.6	1.6 ± 1.0	1.3 ± 1.3	1.9 ± 2.0	0.9 ± 1.5
Lactate, mM/l	5.0 ± 2.7	5.4 ± 2.7	5.7 ± 2.4	5.3 ± 3.0	6.3 ± 5.7
Pyruvate, μM/l	176 ± 87	161 ± 82	148 ± 83	209 ± 234	195 ± 221
Glycerol, μM/l	256 ± 397	260 ± 418	240 ± 273	181 ± 190	284 ± 361
LP ratio	34 ± 39	49 ± 84	74 ± 122	62 ± 116	75 ± 158
LG ratio	5.2 ± 12	8.1 ± 17	18 ± 28	24 ± 41	51 ± 65
Number of samples (*n *= 64 patients)	3,059	1,534	816	153	83
Nonpericontusional					
Glucose, mM/l	2.2 ± 1.5	1.5 ± 1.1	1.4 ± 1.2	1.0 ± 1.0	1.9 ± 2.2
Lactate, mM/l	3.7 ± 1.8	4.4 ± 2.8	5.2 ± 2.7	6.4 ± 2.9	5.0 ± 2.6
Pyruvate, μM/l	162 ± 60	150 ± 64	161 ± 64	170 ± 79	161 ± 110
Glycerol, μM/l	171 ± 207	337 ± 450	414 ± 513	735 ± 763	1,029 ± 907
LP ratio	23 ± 9.0	31 ± 24	36 ± 30	42 ± 23	36 ± 20
LG ratio	4.5 ± 9.8	8.2 ± 20	12 ± 24	16 ± 11	10 ± 12
Number of samples (*n *= 26 patients)	903	570	220	34	4

^a^Data are binned to levels of intracranial pressure (ICP), including the lactate:pyruvate (LP) and lactate:glucose (LG) ratios, from 90 catheters (90 patients), in pericontusional and nonpericontusional locations. Data are expressed as means ± standard deviation (SD).

**Table 2:** Pooled microdialysis data and cerebral perfusion pressure^a^

				CPP, mmHg			
**Pericontusional**	**< 40**	**40 to 50**	**50 to 60**	**60 to 70**	**70 to 80**	**80 to 90**	**> 90**

Glucose, mM/l	0.5 ± 0.7	1.0 ± 1.2	1.5 ± 1.4	1.7 ± 1.4	2.0 ± 1.6	2.2 ± 1.6	2.1 ± 1.4
Lactate, mM/l	11 ± 6.3	5.0 ± 3.6	5.3 ± 2.6	5.3 ± 2.7	5.2 ± 2.6	5.0 ± 2.5	4.8 ± 2.8
Pyruvate, μM/l	253 ± 190	263 ± 348	147 ± 85	166 ± 83	171 ± 89	177 ± 85	178 ± 89
Glycerol, μM/l	307 ± 312	166 ± 213	258 ± 303	240 ± 374	262 ± 430	247 ± 377	315 ± 422
LP ratio	45 ± 20	55 ± 117	61 ± 105	46 ± 78	45 ± 74	36 ± 49	28 ± 14
LG ratio	105 ± 70	31 ± 43	15 ± 25	8.9 ± 19	5.6 ± 12	3.6 ± 3.4	4.6 ± 9.4
Number of samples (*n*₌ 64 patients)	45	67	825	1,992	1,576	646	388
Nonpericontusional							
Glucose, mM/l	1.2 ± 0.7	1.6 ± 0.9	2.0 ± 1.3	1.7 ± 1.4	1.7 ± 1.5	2.1 ± 1.6	2.6 ± 1.4
Lactate, mM/l	4.8 ± 1.1	4.1 ± 1.6	3.8 ± 2.1	4.1 ± 2.7	4.4 ± 2.9	4.1 ± 2.2	4.3 ± 1.5
Pyruvate, μM/l	162 ± 52	151 ± 58	155 ± 73	155 ± 61	161 ± 62	165 ± 58	188 ± 57
Glycerol, μM/l	309 ± 410	327 ± 513	356 ± 462	294 ± 415	190 ± 242	186 ± 213	144 ± 170
LP ratio	36 ± 25	28 ± 8.7	25 ± 14	28 ± 24	29 ± 24	25 ± 9.4	23 ± 7.1
LG ratio	5.2 ± 2.6	4.6 ± 6.4	4.5 ± 8.4	8.2 ± 19	9.6 ± 22	5.6 ± 11	3.7 ± 5.9
Number of patients (*n*₌ 26 patients)	47	130	372	571	375	142	65

^a^Data are binned to levels of cerebral perfusion pressure (CPP), including the lactate:pyruvate (LP) and the lactate:glucose (LG) ratios, from 90 catheters (90 patients) in pericontusional and nonpericontusional locations. Data are expressed as means ± standard deviation (SD).

### Longitudinal trends of MD

**Figure 3:**
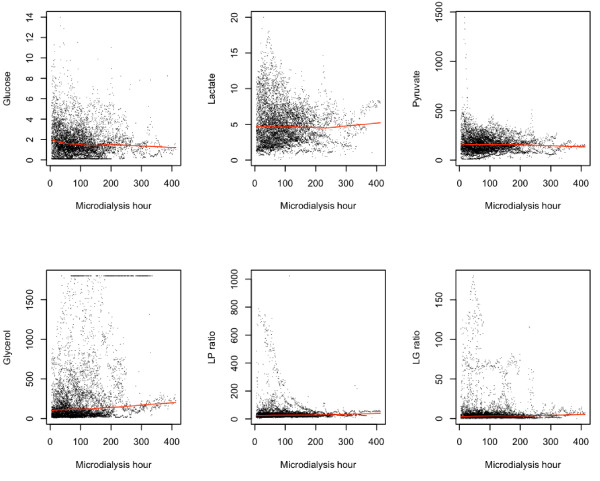
**Trends in cerebral microdialysis (MD) markers**. A locally fitted regression (lowess) was applied to the MD data from the time of MD catheter insertion to visualize trends and evaluate any potential need for detrending in further analyses (90 catheters, 90 patients). Lactate:pyruvate (LP) ratio, lactate:glucose (LG) ratio.

MD data from all patients are plotted from the time of catheter insertion with a lowess regression (Figure 3), showing no clear trends motivating detrending of the data prior to analysis. The same applies for the nonpericontusional catheters separately (not shown). This was confirmed as detrending data after second- and third-order polynomial fits did not enhance later ICP or CPP predictions.

### Autocorrelation

**Figure 4:**
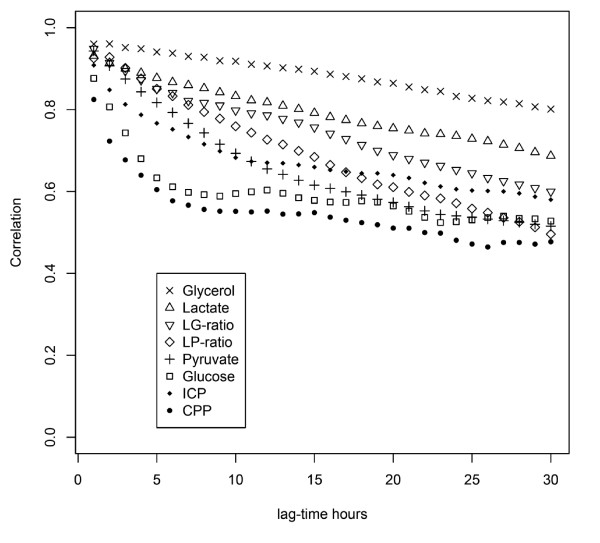
**Autocorrelations of microdialysis (MD) parameters, intracranial pressure (ICP) and cerebral perfusion pressure (CPP)**. These data are shown for up to 30 hours. Variables are highly autocorrelated. Glucose and CPP are the least autocorrelated and thus the most dynamic variables. Lactate:pyruvate (LP) ratio, lactate:glucose (LG) ratio. MD data from pericontusional catheters.

MD, ICP and CPP are all shown to be highly autocorrelated (Figure 4), where CPP and glucose exhibit the least autocorrelation and glycerol exhibits the highest. The degree of autocorrelation is found to extend so far over time that individuals are identifiable. This is seen with linear regression analyses using subject identities as sole explanatory variables. Here subject identity alone is found to explain the variance of MD to 52% to 75% (glucose *r*^2 ^= 0.52, lactate *r*^2 ^= 0.75, pyruvate *r*^2 ^= 0.63, glycerol *r*^2 ^= 0.62, LG ratio *r*^2 ^= 0.69, LP ratio *r*^2 ^= 0.54) in 90 patients. These results indicate that the dominant information in MD using these markers reflects long-term processes (on the order of days) and that all other factors dynamically affecting MD must consequently share the remaining unexplained variance.

### Cross-correlations of MD and ICP and/or CPP

**Figure 5:**
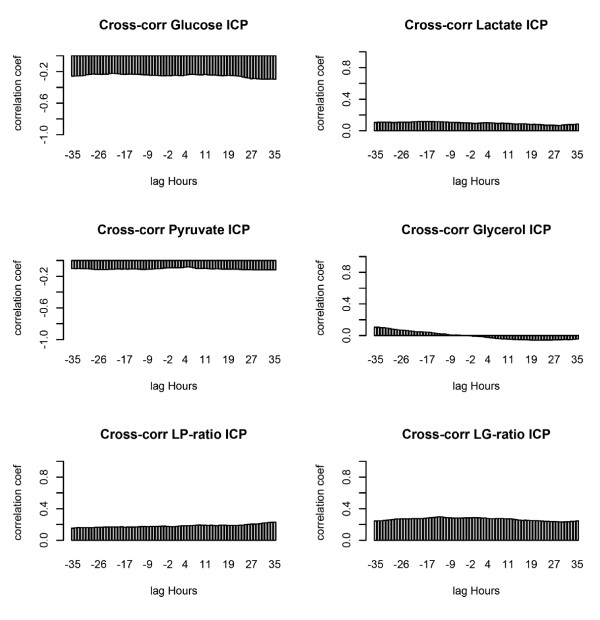
**Cross-correlations of microdialysis (MD) markers and intracranial pressure (ICP)**. Correlations are shown shifted (lag hours) from -35 to +35 hours around MD sample time. The lactate:glucose (LG) ratio is most highly correlated with ICP, but no clear peak time relation is seen. Lactate:pyruvate (LP) ratio. MD data from pericontusional catheters.

**Figure 6:**
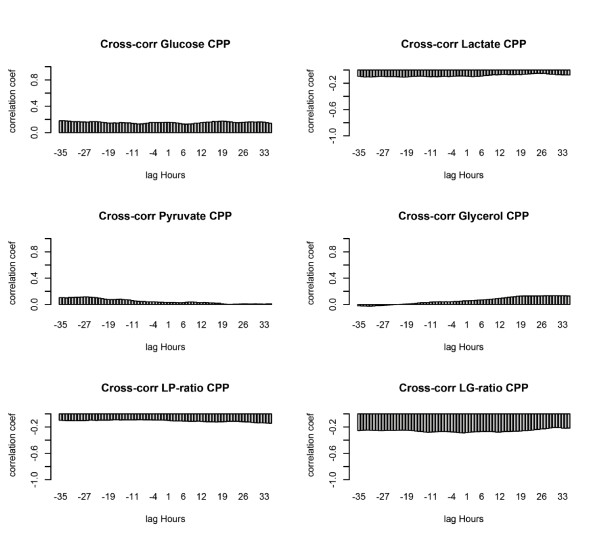
**Cross-correlation of microdialysis (MD) markers and cerebral perfusion pressure (CPP)**. Correlations are shown shifted (lag hours) from -35 to +35 hours around MD sample time. The lactate:glucose (LG) ratio is most highly correlated with CPP, but no clear peak time relation is seen. Lactate:pyruvate (LP) ratio. MD data from pericontusional catheters.

**Figure 7:**
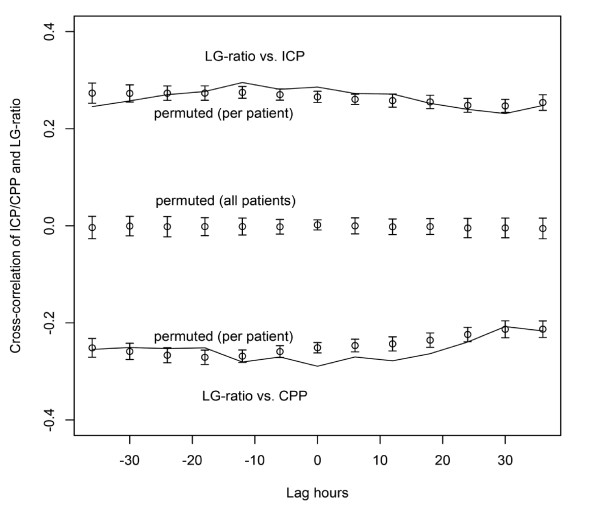
**Effects on cross-correlation of randomly permuting microdialysis (MD) data per patient**. Cross-correlations of the lactate:glucose (LG) ratio (lines) vs. intracranial pressure (ICP) and cerebral perfusion pressure (CPP) are shown as in Figures 5 and 6 shifted (lag hours) from -35 to + 35 hours around MD sample time. In addition, the analyses are performed with randomly permuted (scrambled) MD data per subject (dots), including a Monte Carlo-derived confidence interval (0.95). The correlations are little affected by scrambling subject MD hours in relation to their own ICP and/or CPP. The correlations above (ICP) and below (CPP) that of the per-subject scrambled data represent the added information in excess of a subject's LG ratio mean. A control randomly permuting data from all patients shows zero correlation as expected.

In contrast to the strong autocorrelations presented above, the associations between MD variables and ICP and/or CPP are weak, explaining at most 9% of variance (Figures 5 and 6). These weak correlations are also seen to be similar at all time offsets (lag hours) between MD and ICP and/or CPP and are thus independent of when MD and ICP and/or CPP are sampled in relation to each other. This strongly suggests that it is the mean patient MD values, which are predominately related to mean subject ICP and/or CPP values. This is conclusively tested by randomly permuting (scrambling) the MD time series per subject. Correlations were then found substantially unaltered for all MD markers, ratios and both catheter locations. An example of this is shown for the strongest cross-correlations: those of the LG ratio and ICP and/or CPP from pericontusional catheters (Figure 7). A Monte Carlo-derived confidence interval is applied after repeated random permutations of MD data per subject. A control randomly permuting all the data (also between subjects) gives the expected levels of near-zero correlations.

In the aggregate, these analyses convincingly indicate that the dominant information in univariate correlations of MD values and ICP and/or CPP are weak and that there is little information in the (ordered) time series in excess of data means. The relations between MD and ICP and/or CPP thus appear more related to the individual patient than to any dynamic relationship that can be followed during MD monitoring. Consequently, mean per-patient correlations were also assessed in further analyses (see mean data analysis of MD).

### Multivariate correlations of MD and ICP and/or CPP

Composite patterns of MD variables may be more related to ICP and/or CPP than to individual MD variables. Therefore, multivariate analyses of MD toward ICP and/or CPP were performed with two methods: artificial neural networks and mixed models.

The motivation for using adaptive nonlinear regression methods (such as ANNs) is that they can adjust to potential nonlinear relationships that can exist in limited regions of data (for example, that CPP could be related to an increased LP ratio and low glucose, but only under 50 mmHg). This nonlinearity would not be modeled in a linear regression. A cross-validation procedure (repeatedly leaving out patients from model development and using them to evaluate models) is crucial so that ANNs do not "learn" patients, but instead model the underlying general relationship between MD and ICP and/or CPP.

The results of these analyses are presented in Table 3 as the correlation coefficient between true and predicted ICP and/or CPP, using MD parameters as explanatory variables. Multivariate associations between MD and ICP and/or CPP were found to be weak in both pericontusional and nonpericontusional catheter locations. ICP may be more related to MD in nonpericontusional and CPP in pericontusional positions. (The negative correlation in models designed to make positive predictions is interpreted as "even worse.") Again, there is a clear indication that permuting (scrambling) subject data hours has limited effects on predictions.

**Table 3:** Correlation coefficients of true vs. predicted intracranial pressure (ICP), and cerebral perfusion pressures (CPP), from a non-linear multivariate analysis method (artificial neural networks)^a^

	Pericontusional	Nonpericontusional
ICP	0.165 ± 0.013	0.372 ± 0.019
Permuted	0.129 ± 0.016	0.265 ± 0.021
CPP	0.134 ± 0.018	-0.143 ± 0.020
Permuted	0.059 ± 0.021	-0.111 ± 0.030

^a^ICP, intracranial pressure; CPP, cerebral perfusion pressure. Microdialysis markers (glucose, lactate, pyruvate and glycerol) and ratios (LP ratio and LG ratio) were used as explanatory variables predicting mean ICP/CPP (0 to 60 minutes prior to microdialysis (MD) sample time). In addition, correlations were analyzed after randomly permuting (scrambling) MD data per subject in relation to ICP/CPP to access what remained of correlations if no longer serially related. Negative correlation (modeling positive prediction) is interpreted as "even worse." Data are presented as means ± standard deviation (SD). SD values represent the variation of artificial neural network solutions from 200 separate runs with random initializations.

Linear mixed effects models allow for multivariate analyses of grouped data structures. The dependency of intersubject data can be analyzed as a random effect. The fixed effect (subject-independent effect) between MD and ICP and/or CPP can then be evaluated. In addition, an intrasubject data structure (such as strong correlations and dependency of near lying MD hours) can be introduced. Linear mixed effects analyses were performed with MD markers and ratios as explanatory variables and ICP or CPP as predicted variables. Pericontusional and nonpericontusional data were analyzed separately. Data from adjacent MD hours were, in congruence with the autocorrelation analyses, highly correlated (0.77 to 0.83) in all groups. In addition, these analyses identified glucose and LG ratio as being significantly related (*P*< 0.05) to both ICP and CPP in pericontusional locations, and LP ratio was found to be significantly related to ICP in both the peri- and nonpericontusional positions. There were no significant correlations between MD and CPP in nonpericontusional positions. However, despite these quoted significance values, regression coefficients were small and extreme changes of MD marker values predicted minor changes in ICP and/or CPP, indicating a generally weak association between MD and ICP and/or CPP.

In summary, these analyses also indicate that the correlations between MD and ICP and/or CPP are weak when taking into account the correlated nature of intrasubject data. The main strength of correlation is shown, again, to be related to subject data means. We identified no apparent multivariate and/or nonlinear information that appreciably strengthened correlations.

### Mean data analysis of MD vs. ICP and/or CPP

Regression analysis of mean (per subject) values of MD markers vs. ICP and/or CPP was performed. Here significances were tested against the number of patients (mean of each patient's whole monitoring period) instead of the number of MD samples.

The significance levels in these analyses mirrored those of the mixed model analyses, and the strongest relationships were found between ICP and glucose in pericontusional tissue (*r*^2 ^= 0.16) and LP ratio in nonpericontusional tissue (*r*^2 ^= 0.15). Multivariate regression did not enhance *r*^2 ^values. An automated search for alternative cutoffs of ICP and CPP that could better represent the data (stronger correlations) was performed, but no other representation (hours or percentages of monitoring under or over cutoffs) strengthened correlations, as compared to hourly means.

### Differenced and by-subject normalization of MD data vs. ICP and/or CPP

The results presented so far strongly indicate that the dominant processes affecting MD values follow an appreciably longer time span than (the common) hourly sampling. Differentiated (that is, Δ) values could possibly reveal associations of short-term effects of ICP and/or CPP superimposed on longer trends of MD. Thus, differentiated MD was analyzed toward ICP and/or CPP and differentiated ICP and/or CPP. In these uni- and multivariate analyses, significant changes were found for CPP in pericontusional catheters, but no *r*^2 ^values were greater than 0.002.

In contrast to absolute levels of MD, the particular location of a MD catheter could exhibit a baseline characteristic, and relative changes could be related to ICP and/or CPP. MD was therefore analyzed toward ICP and/or CPP after normalization (per-subject) of the data. These analyses identified statistically significant but weak multivariate correlations (maximum *r*^2 ^= 0.069) for ICP in nonpericontusional catheters. All other analyses had *r*^2 ^< 0.020 between MD and ICP and/or CPP in both catheter locations.

In summary, the two methods exploring relative changes and levels of MD in relation to ICP and/or CPP indicate only weak associations.

### MD, ICP and CPP vs. outcome

**Figure 8:**
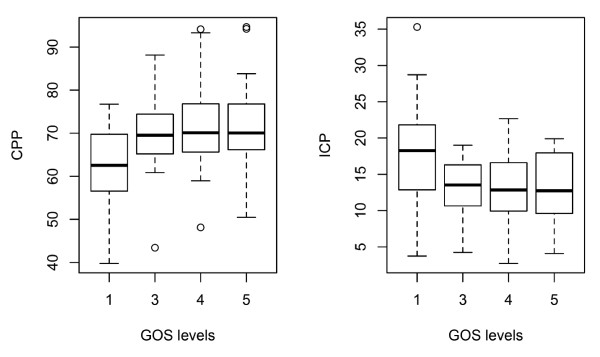
**Glasgow Outcome Scale (GOS) score vs. cerebral perfusion pressure (CPP) and intracranial pressure (ICP)**. Significant differences in means (per subject) were seen for CPP (*P *= 0.014) and ICP (*P *= 0.021) when compared to GOS levels (one-way analysis of variance). The box-and-whisker plot indicates the median, the lower and upper quartiles (boxes) and the 1.5 * interquartile range (whiskers). Outliers (outside whiskers) are indicated as circles.

A reduction to mean values per subject the examination of MD and ICP and/or CPP with respect to other global (per-subject) parameters, such as outcome. There were significant differences in subject means for CPP (*P *= 0.014) and ICP (*P *= 0.021) as related to GOS levels (Figure 8). In contrast, there were no significant differences for any MD means, MD ratios or increased LP ratios (hours or percentage of monitoring time) (*P *= 0.14 to 0.96) as compared to GOS score in the full data set or in subgroups of peri- and nonpericontusional catheters. Significant findings (*P *< 0.05) were unchanged with both Kruskal-Wallis ranked and one-way ANOVA analyses. Excluding the subject with only a discharge GOS score did not alter significance values.

## Discussion

In this study comprising more than 7,350 hourly samples of complete MD sets from 90 patients, we have performed an extensive search with several types of statistical and computer-based linear and nonlinear pattern recognition methods to explore the relationship between ICP, CPP and the commonly used MD markers in TBI monitoring. The main finding is that despite much of the data indicating highly perturbed metabolism, the relationships between MD and ICP and/or CPP are weak. This suggests that factors other than these pressure and/or surrogate flow variables may be dominant causes of perturbations in the clinical TBI setting. In contrast, intrasubject correlations (autocorrelation) of MD are high for all MD parameters and ratios, even up to 30 hours. In fact, these autocorrelations are so extended in time that subject identities alone explain 52% to 75% of MD variable variance. This indicates that the dominant patterns of MD seen in TBI (with the studied variables) are protracted, reflecting processes that change over days or longer. This leaves limited unexplained variance to be shared among other variables that have been shown to affect MD values during monitoring, such as hyperventilation [26], meningitis [27], temperature [28] and seizures [29]. Importantly, this applies to catheters in both (CT-defined) pericontusional and nonpericontusional locations. In contrast to long-term associations of MD, short-term (differentiated) associations of MD, though significant for CPP in pericontusional tissue, explain only up to 0.2% of variance. These results may not be harmonious with the expectations of MD as a dynamic and interpretable online monitor of ischemia and/or hypoxia in TBI. In addition, a significant relation was found between CPP and/or ICP and GOS score, but this could not be confirmed for MD and GOS score.

MD is commonly sampled once per hour in the NICU. The objective is to monitor short-term changes and more long-term trends. Short-term changes have focused primarily on potentially ischemic and/or hypoxic interpretations of the data, where increased LP ratios and low glucose have often been implicated as being local ischemic and/or hypoxic metabolic responses [1]. The traumatic border zone has been recognized as distinctly different from the ischemic penumbra as well as regionally heterogeneous [30-32]. The interpretation of more long-term patterns of metabolic perturbation have received less focus, but increased LP ratios have also been linked to different causes of altered oxygen utilization in TBI, as opposed to oxygen delivery. These include oxygen diffusion barriers [16], mitochondrial dysfunction [15,33] and increased metabolism of glucose [34]. In addition, irreversibly damaged (posthypoxic) but reperfused regions may also display extended periods with MD of ischemic and/or hypoxic character [35]. Alternative interpretations of lactate, pyruvate and LP ratios in TBI have therefore been postulated [11], and more complex supply-and-demand relations of these parameters under nonischemic conditions have also been identified [36]. Moreover, MD may also be influenced by static parameters such as catheter placement in gray or white matter [37], genetics [38] and patient sex [39]. Our study strongly indicates that the dominant information content in MD of TBI patients are that of long-term patterns, which is reflected in the strong autocorrelations, and that MD can so highly be explained by subject identities. This includes the LP ratio, which is also seen to be highly autocorrelated. The significant but weak cross-correlations between MD and ICP and/or CPP are also seen to be predominantly caused by long-term perturbations, as the correlations are largely unaffected by how subject MD data hours are serially related to ICP and/or CPP hours. In addition, the MD response to CPP and/or ICP changes is variable even in ranges that are by consensus considered unsafe. This may lead clinicians to question the current value of hourly sampling of MD in clinical TBI monitoring in the absence of known cause-and-effect relationships and points to a need for more reliable interpretations of pathological values for clinical use. We suspect that to differentiate patterns displaying similar levels, and possibly with different etiologies, one needs also to better include temporal relations of MD patterns.

The effect of CPP and/or ICP on local and global blood flow in TBI is complex [9], and the effects on MD have been found to be variable. Extreme ranges of ICP and/or CPP have been shown to have predictable effects on regional MD [7], and Nordström et al. [8] identified MD changes related to CPP < 50 mmHg and > 70 mmHg. In contrast, CPP augmentation has been shown to increase cerebral blood flow with positron emission tomography (PET), but not to translate to predictable changes in regional chemistry as seen with MD [40]. In addition, increased LP ratios in pericontusional tissue have been shown to be independent of CPP [20]. Our study distinguishes long-term from short-term relationships between MD and ICP and/or CPP. We have used multiple analytical techniques to assess our data, and the findings are in basic congruence. Despite a weak correlation, CPP is found to be related to MD exclusively in pericontusional tissue, whereas ICP is related to MD in both peri- and nonpericontusional tissues. In contrast to long-term relations, differentiated short-term values were exclusively related to CPP in pericontusional locations, but with < 0.2% explained variance. Moreover, we cannot confirm the findings of Belli et al. [41], who found that increased LP ratios preceded increased ICP. Multivariate analyses in our study also suggest that the composite strongest association is found between MD and ICP in nonpericontusional data, which is reasonable as this catheter placement may represent a more global monitor. Glucose is identified as the most dynamic marker and is least autocorrelated. In the aggregate, we can identify several expected and previously known relations between CPP and/or ICP and MD, but we conclude that the explained variance is such that MD perturbations must have other main causes.

A few studies have related MD to GOS score. Patient outcome was earlier been found to be related to high MD potassium [42], increased lactate and low glucose [43], persistent low glucose [44,45] and increased glutamate [46,47] levels, but variable for glycerol [48,49]. Recently, *N*-acetylaspartate sampled by MD has also been implicated as a marker of outcome [50]. That MD is so highly subject-related motivates comparisons of mean (per subject) MD and GOS score. We found that GOS score was significantly related to CPP and ICP, but not to any separate MD marker level (glucose, lactate, pyruvate, or glycerol) or MD ratio. This indicates that the extremely local nature of this monitoring method may portray information that does not necessarily translate to a total patient situation.

Consequently, to our knowledge, there exists no current interpretation of absolute, relative or trend data of the current common MD variables that can be strongly and consistently related to explanatory variables, such that it lends evident support to clinical decision-making, a prerequisite for any monitoring system. In addition, although the distinction of peri- and nonpericontusional catheter locations appears to provide different information, possibly on the basis of different metabolic processes, the identification of pericontusional tissue may be uncertain on CT scans [31]. Using MD as an alert signal (toward normality-good or away from normality-bad) [11] appears logical but must be accompanied by identifiable cause-and-effect relations on the basis of which to steer interventions. As yet, this information is, to our knowledge, lacking for MD.

A potential weakness of the study is that the caregivers were not blinded to the MD data. During periods when MD displayed possible true ICP and/or CPP dependencies, these have been identified and acted on. We do not believe this to be the case, as doctors' responses to pathological MD values vary greatly. Moreover, no standardized treatment algorithms were suggested during this study or in the literature. In addition, resistance governs the relation between CPP and flow. This is affected by autoregulation, which we have no measure of globally or locally. An additional weakness is that we have no direct measure of tissue hypoxia with which to validate the absence or presence of such in relation to MD values. A further weakness is that we have not related MD to interventions such as ventricular drainage of CSF, additional increase of CPP, Pentothal infusions or decompressive craniotomies. Therefore, we cannot exclude that such measures could have had an impact on MD in our study.

## Conclusions

In this study we have used extensive data mining employing linear and nonlinear techniques to establish the relationship between MD and ICP and/or CPP, parameters that are expected to affect local blood flow and thus, to some extent, oxygen delivery. Our results indicate that, despite much data indicating highly perturbed metabolism, MD shows little correlation to ICP and CPP within the constraints of these parameters in the NICU. In addition, ICP or CPP predictions were not meaningfully improved when catheters were placed in CT-defined pericontusional locations versus nonpericontusional catheters. In contrast, MD is strongly autocorrelated, and variance is highly explained by intrasubject data correlations, indicating that the dominant processes followed with MD in TBI are long-term over a period of days. In addition, short-term changes of MD are seen to exhibit extremely weak associations with ICP and/or CPP. For MD to find a clear place in clinical TBI monitoring, it is essential that we establish a better understanding of the causes for long-term metabolic perturbations, and seek additional dynamic markers of tissue distress [21,50,51]. More studies relating local MD and MD changes to other measures of tissue hypoxia, such as local brain tissue oxygenation monitoring and PET are needed. Cerebral microdialysis remains as yet the only way to repeatedly sample local one-line extracellular brain chemistry and is as such an important tool in TBI research.

## Competing interests

The authors declare that they have no competing interests.

## Authors' contributions

DWN was involved in the design of the study, analyzed all data and drafted the manuscript. BT was involved in data analysis. RMM analyzed data with special involvement in machine learning methods. HN evaluated all CT scans for catheter placement. AH analyzed the data with special involvement in time series analyses. AR participated in design of the study and manuscript preparation. MW participated in the design of study and helped in manuscript preparation. BMB was in charge of the patient database, the design of the study and manuscript preparation. EW was responsible for the project, the design of the study and manuscript preparation. All authors read and approved the final manuscript.

## Pre-publication history

The pre-publication history for this paper can be accessed here:

http://www.biomedcentral.com/1741-7015/9/21/prepub
